# Prior cocaine use disrupts identification of hidden states by single units and neural ensembles in orbitofrontal cortex

**DOI:** 10.1101/2025.03.28.645972

**Published:** 2025-03-31

**Authors:** Wenhui Zong, Lauren E. Mueller, Zhewei Zhang, Jingfeng Zhou, Geoffrey Schoenbaum

## Abstract

The orbitofrontal cortex (OFC) is critical to identifying task structure and to generalizing appropriately across task states with similar underlying or hidden causes ^1–6^ . This capability is at the heart of OFC’s proposed role in a network responsible for cognitive mapping ^7,8^ , and its loss can explain many deficits associated with OFC damage or inactivation ^9^ . Substance use disorder is defined by behaviors that share much in common with these deficits, such as an inability to modify learned behaviors in the face of new or even anecdotal information about undesired consequences. One explanation for this similarity would be if addictive drugs impacted the ability of OFC to recognize underlying similarities – hidden states – that allow information learned in one setting to be used in another. To explore this possibility, we trained rats to self-administer cocaine and then recorded single unit activity in lateral OFC as these rats performed in an odor sequence task consisting of unique and shared positions. In well-trained controls, we observed chance decoding of sequence at shared positions and near chance decoding even at unique positions, reflecting the irrelevance of distinguishing these positions in the task. By contrast, in cocaine-experienced rats, decoding remained significantly elevated, particularly at the positions that had superficial sensory differences that were collapsed in controls across learning. A tensor component analysis showed that this effect of reduced generalization after cocaine use also extended across positions in the sequences. These results show that prior cocaine use disrupts the normal identification of hidden states by OFC.

## Results

To examine the potential impact of cocaine use on the identification of hidden states by OFC neurons, we used a go, no-go odor discrimination task (Figure 1A). This task, used previously to record in OFC [10], featured 6 odors arranged in two 4-odor sequences, labeled as S1 and S2 (Figure 1B). These sequences had unique odors at the start and end positions (P1 and P4) and shared odors at the two positions in the middle, where the sequences overlapped (P2 and P3). The experiment was conducted over a 4-month period consisting of several phases (Figure 1C). Initially, naive rats underwent a four-week training on the "figure 8" task. In the subsequent week, rats underwent jugular catheterization surgery and were given time to recover. For the following two weeks, rats were trained to self-administer either sucrose (10% wt/vol; $n$ = 3) or cocaine (0.75 mg/kg/infusion; $n$ = 3) (Figure 1D), using procedures similar to those known induce incubation of craving [11]. The subsequent three weeks were dedicated to electrode implantation surgery in OFC and post-surgical recovery. Two additional weeks were then allocated for reminder training on the odor task. Finally, *in vivo* recordings were obtained during the last four weeks while the rats performed the odor task.

During these recording sessions, rats in both groups displayed consistently high levels of discrimination performance across all positions in the odor sequence (Figure 1E). Differences in trial initiation latencies were observed (Figure 1F), suggesting that the rats in both groups utilized the predictable odor sequence to anticipate the availability of reward not only in the current trial but also in subsequent trials. Importantly, there were no significant effects of cocaine use on these performance measures. During these sessions, we recorded a total of 1,699 units in the lateral OFC of thee rats in the cocaine group and 2,182 units from three rats in the control group (Figure 1G).

*Prior cocaine use disrupts identification of hidden states across position pairs*

We investigated whether cocaine use affected the ability of single units and ensembles in the OFC to discriminate between positions in the task, starting with comparisons between the same positions on sequences S1 and S2. Neuronal selectivity on trials at comparable positions in the two sequences was calculated for each trial epoch, and the results showed that the proportion of selective units was very low—essentially at chance during trials at P2 and P3—before increasing at the end of P3 and into the odor sampling periods of P4 and P1 (Figure 2A, top panel). This pattern reflected compression of positions in the central arm of the figure-8 maze, where the two sequences were identical, and discrimination of positions on the outer arms of the maze, where the odors differed. Notably, however, even for P4 and P1, most epochs within the trial showed near chance levels of selectivity in controls (Figure 2A, top panel), while neurons recorded in cocaine rats maintained significantly higher levels of selectivity (compared both to controls and chance) at the end of P3 and during most of the epochs in P4 when compared directly between the two groups (Figure 2A, bottom panel and Figure 2B).

These patterns were even more pronounced in an ensemble decoding analysis (Figure 3A). Decoding accuracy was above chance for most epochs at positions P1 and P4 and at the end of the trial and into the ITI period at P3, and while this pattern was true in both groups, the decoding accuracy was significantly higher in the cocaine group in nearly all epochs. This difference between groups was even more evident when average decoding across epochs at each position was directly compared between the two groups (Figure 3B). Thus, both single unit and ensemble activity in lateral OFC in cocaine-experienced rats compressed meaningful task epochs less than in sucrose-trained controls.

The effect of cocaine was similar to the effect of less training. This is evident in a comparison to data from OFC in rats in a prior study using the same task [10], in which there was much less training prior to recording (6 weeks of odor task training in total plus 2 weeks of self-administration in this study vs. ~3 weeks of odor task training in the prior study). OFC neurons recorded in these rats revealed preserved decoding relative to the over-trained controls in the current study, decoding that was similar to that in the cocaine rats (Figure 3C). Similar effects of training were also evident in OFC neurons recorded in a more complex odor sequence task, also reported previously [12], where the preservation of decoding between positions was linked to behavioral relevance (Figure S1). Thus, neural activity in OFC evolves during learning, to identify the underlying hidden states that define behavioral relevance. This refinement or its specificity is disrupted by prior cocaine use.

*Prior cocaine use disrupts identification of hidden states across all positions*

While the planned analyses conducted above show that prior cocaine use was associated with preserved discrimination of incidental information about task positions normally compressed by rats performing this task, they do not address whether this is a general effect or whether it only impacted direct comparison of the position pairs highlighted by the analyses. That is, did neurons in the cocaine-experienced rats maintain information about these positions because of the idiosyncratic features of our task, or is there a general effect of cocaine on the ability of the OFC to register common features and underlying causes. To get at this more general question, we utilized tensor component analysis (TCA) to identify the dominant factors shaping neural activity across units, epochs, and trials in our task. This analysis provides a less constrained, more hypothesis-agnostic approach to our question since it looks for patterns across all of these factors in the design. If cocaine is causing a failure of generalization more broadly, then we would expect loss of power in dimensions across positions and not just within position pairs.

The results for this analysis are shown in Figures 4A-B. Neural variance was partitioned into components, one per row. The analysis shown was constrained to 10 components, as this number produced the most consistent and reliable results compared to other choices (Figure S2-4). For each group, the characteristics of each component are visualized by the contributing neuron weight (left), temporal dynamics within a trial (middle), and amplitude of such dynamics across trials organized by positions (right). Thus, to discuss one example, the top row in the control group shows that the first component was broadly distributed across neurons (left panel), expressed most strongly in the later trial epochs (middle panel), and exhibited low amplitude at P1 and P4 and high at P2 and P3 (right panel). Further, it was higher in P3 than P2 and also showed similar patterns across trials within each of these position pairs (right panel).

After aligning these component patterns between groups based on their temporal factors, we observed different patterns in trial amplitudes reflecting differences in positional representations (right columns). In controls, most of the trial factors distinguished between the position pairs (rows 1–3, 6, 7, 8, 10). These factors distinguished rewarded from non-rewarded position pairs, in some cases further distinguishing particular position pairs within each category (rows 2,3,7,8) and/or showing effects of trials (rows 2,3). However, none showed differences between positions or trials inside a pair. This result is consistent with findings in Figure 2 and 3, which showed that both individual units and ensemble responses in control rats collapsed the positions within each pair. However, controls also had three additional factors that appeared to generalize not just across positions inside a pair but rather across all positions (rows 4, 5, 9). These factors were identical across all 8 positions and two (rows 5 and 9) showed identical changes across trials within each position. This indicates a significant tendency in neural activity in OFC to represent commonalities across all 8 positions dynamically across time in a session; this global generalization goes beyond that illustrated in our planned comparisons in Figures 2 and 3.

The trial factor patterns in the cocaine-experienced rats were substantially different; all of the 10 factors distinguished rewarded versus non-rewarded position pairs, and the position-general factors evident in controls (rows 4, 5, 9) were absent from activity in the cocaine rats. This dichotomy is consistent with reduced compression evident in our planned comparisons in Figures 2 and 3 and further points to diminished generalization across task features and a heightened emphasis on differences across positions in the task, which presumably reflects the different values imparted by the sequences.

To quantify the differences in Figures 4A–B, we used mutual information (MI) to measure the information available in each factor—particularly factors 5 and 9 about position and trial. Consistent with the description above, we found lower MI values for position in controls at these components compared to the cocaine group (Figure 4C) and higher MI at the same components for trial or temporal information (Figure 4D). Moreover, similar results were observed across varying numbers of components, with the significant difference between the two groups remaining consistent regardless of the number of components selected (Figure S3-4).

### Discussion

These results are consistent with a cocaine-induced disruption of the normal identification of underlying hidden states by OFC [1-6]. Specifically, in the current task, OFC representations normally evolve with training to compress or generalize similar positions in the two sequences, even when external sensory information—the odor cues—differ. Cocaine-experienced rats failed to show this normal compression of irrelevant information, instead discriminating these position pairs at higher rates, similar to encoding observed in rats early in learning. Additionally, this failure to generalize was also evident in a TCA analysis, where factors generalizing across different positions were prominent in control data but entirely absent in data from cocaine-experienced rats.

The loss of this normal function of OFC is relevant to addiction, since it suggests that some addictive drugs, at least the psychostimulants, cause fundamental and long-lasting changes in how prefrontal areas process task-related information. These effects are consistent with prior studies showing changes in OFC function after drug use [13,14], however they extend this work to show a specific effect on generalization or the ability of OFC neurons to collapse trivial external information to encode underlying causes that different situations have in common. A loss of this ability would have wide ranging effects [15], but it might particularly disrupt the mobilization of learning from other settings to counteract or diminish drug-seeking behaviors. For instance, new or anecdotal consequences of drug use learnt in separate contexts would not be as effectively deployed to affect behavior in drug-taking contexts. Similarly therapeutic approaches designed to extinguish drug-seeking would also generalize more poorly outside of the clinical setting. The current data provides a neurophysiological correlate, induced by drug-use, that could underlie such well-known effects.

## Figure Captions

**Figure 1: Figure 8 odor sequence task, cocaine self-administration, and behavior**

(**A**) Schematic representation of the trial events in the odor-sequence task. The initiation of each trial was indicated by the illumination of two overhead houselights. After poking into the central odor port and sampling the presented odor, rats had the option to respond with a 'go' to receive a sucrose reward or a 'no-go' to avoid a prolonged inter-trial interval. (**B**) The six odors were grouped into two sequences, S1 and S2, each consisting of four odor positions (P1–P4). The sequences alternated in a "figure eight" pattern. The numbers at each position represent odor identities, and the blue/red symbols (+/-) indicate rewarded and non-rewarded trials. (**C**) Timeline of the experimental procedure. (**D**) The number of active (solid lines) and inactive (dashed lines) lever presses during sucrose self-administration sessions (upper; $n = 3$) and cocaine self-administration sessions (lower; $n = 3$). (**E**) Percentage of correct responses (% correct) for each trial type during single-unit recording sessions. Blue indicates trial types with rewards, while red indicates trial types without rewards. Error bars represent standard errors of the mean (SEMs). (**F**) Poke latency, measured as the time from light onset to odor port entry. Error bars represent SEMs. (**G**) Reconstruction of recording sites, with red squares indicating the locations of electrodes.

**Figure 2: Discriminating sequences S1 versus S2 at single-unit level**

(**A**) Percentage of neurons selective at each position in S1 versus S2 in the Sucrose (upper panel) and Cocaine (lower panel) groups. The selectivity was calculated for different task epochs ["ITI-a", "Light" , "Poke", "Odor" , "Unpoke" , "Choice" , "Outcome" , "Post-Out" , "ITI-b"] for all four positions (one-way ANOVA). Epochs within the red dashed boxes indicate selectivity exceeds a 95% confidence interval (CI) (Sucrose: $\chi^2$'s > 5.4; $p$'s < 0.02; Cocaine: $\chi^2$'s > 4.0; $p$'s < 0.05; Chi-squared test). The $p$-values were not corrected, and the dotted lines represent the chance level of selectivity according to this criterion. Notably, rats could discriminate between the two sequences (S1 vs. S2) based solely on current sensory information at two task epochs ["Odor" at P3 and P4; black bars]. At all other task epochs, indicated by gray bars, the discrimination relied on an internal memory of events. (**B**) Line plot showing neuronal selectivity at each position in S1 versus S2 for each neuron in both the Sucrose (black line) and Cocaine (red line) groups. The selectivity was calculated at different task epochs for all four positions. Each asterisk indicates that there are significant differences between the two groups ($\chi^2$'s > 4.8; $p$'s < 0.03; Chi-squared test). *$p < 0.05$; error bars are standard deviations (SDs).

**Figure 3: Cocaine use reduces the ability of OFC to generalize task-irrelevant odor-overlapping sequences.**

(**A**) Decoding accuracy of S1 versus S2 was evaluated within each of the nine task epochs for positions P1 to P4. Error bars represent SDs, and each asterisk indicates that the mean decoding accuracy exceeds a 95% confidence interval calculated using the same decoding process with label-shuffled data. The dotted lines represent the chance level of decoding. The meaning of the black bars is consistent with **Figure 2A**. (**B**) Decoding accuracy of S1 versus S2 at each position was assessed using varying ensemble sizes for the Sucrose (black) and Cocaine (red) groups across all epochs. The Cocaine group demonstrated higher decoding accuracy and reduced generalization of task-irrelevant, overlapping sequences compared to the Sucrose group ($F_{(1,118)} = 16.8$, $p = 7.8 \times 10^{-5}$, $\eta_p^2 = 0.12$; One-way ANOVA). P1, P2, P3, and P4 represent positions 1, 2, 3, and 4, respectively. (**C**) Decoding accuracy of S1 versus S2 at each position was assessed using varying ensemble sizes for the Sucrose group

(black, same data as in **Figure 3B**) and the Less Training group (blue, Zhou et al., 2021) across all epochs. The decoding accuracy in the Less Training group was comparable to that observed in the Cocaine group (**Figure 3B**, red) ($F_{(1,118)}$ = 0.01, p = 0.92, $\eta_p^2$ = 9.0 × $10^{-5}$; One-way ANOVA). Additionally, the Less Training group exhibited significantly higher decoding accuracy than the Sucrose group ($F_{(1,118)}$ = 18.1, $p$ = 4.2 × $10^{-5}$, $\eta_p^2$ = 0.13; One-way ANOVA). P1, P2, P3, and P4 represent positions 1, 2, 3, and 4, respectively. *$p$ < 0.05; error bars indicate SDs.

**Figure 4: Cocaine use impairs the OFC's function in temporal cognition and decreases the generalization of task-irrelevant odor-overlapping sequences**

(**A-B**) Ten components of TCA applied to Sucrose(**A**) and Cocaine(**B**) neuron activity are displayed. Each component consists of a neuron factor (left column), a temporal factor spanning nine events with eight time points per event (middle column), and a trial factor (right column). Temporal factors are grouped as follows: 1–8 (preTrial), 9–16 (Light), 17–24 (Poke), 25–32 (Odor), 33–40 (Unpoke), 41–48 (Choice), 49–56 (Outcome), 57–64 (postTrial1), and 65–72 (postTrial2). The dataset includes eight trial types, each with 40 trials. Trial factors 1–80 and 241–320 represent rewarded (positive) trials, while 81–240 correspond to unrewarded (negative) trials. Trials are further categorized as follows: 1–80 (P1), 81–160 (P2), 161–240 (P3), and 241–320 (P4). These low-dimensional components were extracted from a 10-component model for both the Sucrose(**A**) and Cocaine groups(**B**). In the Sucrose group, components 1, 6, 7, and 10 encode positive trials, while components 2, 3, and 8 encode negative trials. Similarly, in the Cocaine group, components 1, 4, 6, 7, and 10 encode positive trials, whereas components 2, 3, 5, 8, and 9 encode negative trials. Notably, in the Sucrose group, beyond reward-related components, additional components encode aspects of temporal cognition (components 5 and 9), along with early components broadly active across most trials (component 4). (**C**) Plot illustrating mutual information (MI) between across-trial factors and trial types. The Sucrose group exhibits lower MI, particularly at components 5 and 9. A two-way ANOVA confirmed a significant reduction in MI for the Sucrose group ($F_{(1,1360)}$ = 571.6, p = 9.7 × $10^{-106}$, $\eta_p^2$ = 0.3), especially at these components, which showed consistent patterns across all trial types, aligning with the plots in (**A**) of the Sucrose group, indicating a weaker dependency between across-trial factors and trial types. A significant difference was also observed across components ($F_{(9,1360)}$ = 266.3, $p$ = 1.2 × $10^{-292}$, $\eta_p^2$ = 0.64). (**D**) Plot illustrating mutual information (MI) between across-trial factors and time. The Sucrose group demonstrates greater MI, particularly at components 5 and 9, suggesting a stronger dependency between across-trial factors and time. A two-way ANOVA confirmed a significant increase in MI for the Sucrose group ($F_{(1,1360)}$ = 850.1, $p$ = 1.4 × $10^{-145}$, $\eta_p^2$ = 0.38). Additionally, a significant difference was observed across components ($F_{(9,1360)}$ = 176.8, $p$ = 1.4 × $10^{-221}$, $\eta_p^2$ = 0.54). Error bars are SEM.

**Figure S1: The ability of the OFC to generalize task-irrelevant odor-overlapping sequences decreases as learning progresses**

(**A**) Organization of sixteen odors into two pairs of sequences (S1a-S1b and S2a-S2b), with each sequence consisting of six trials or positions (P1-P6). The blue '+' symbol represents rewarded trials, while the red '-' symbol represents non-rewarded trials. Odor identities are represented by numbers 0-15, and arrows indicate transitions between sequences. (**B-C**) The percentage of decoding accuracy was analyzed across different groups with varying training days and ensemble sizes. Significant differences were observed between S1a and S1b across all time periods (days 1–5, days 6–10, and days 11–15) for different numbers of selected neurons ($F_{(2,42)}$ = 11.2, $p$ = 1.3 × $10^{-4}$, $\eta_p^2$ = 0.35). In contrast, no significant differences were found between S2a and S2b ($F_{(2,42)}$ = 0.17, $p$ = 0.84, $\eta_p^2$ = 8.2

$\times 10^{-3}$). Additionally, significant differences were identified between S1 and S2 ($F_{(1,88)} = 8.69$, $p = 4.1 \times 10^{-3}$, $\eta_p^2 = 0.09$). The error bars indicate SDs.

**Figure S2: Error and similarity plots for TCA**

The optimization landscape of TCA may contain suboptimal solutions (local minima), necessitating iterative optimization to minimize a cost function. To evaluate the stability of this process, we generated error and similarity plots. Specifically, we ran the TCA optimization algorithm 100 times for 10 components, each initialized with random conditions, and plotted the normalized reconstruction error across all runs. This approach enabled us to assess whether certain runs converged to local minima with high reconstruction errors. As shown in **Figures S2A-B** (left panels), the error plots indicate that all 100 runs at a fixed number of components produced nearly identical reconstruction errors. Additionally, models with 9 and 10 components exhibited very similar errors. The similarity plot (**Figures S2A-B**, right panel) further quantifies the consistency of TCA models across different component numbers (horizontal axis). For each model, similarity scores were computed relative to the best-fit model with the same number of components, with the lines representing the mean similarity as a function of the number of components. Across all tested component numbers, the 100 repeated runs per component showed substantial overlap and consistently yielded similarity scores above 0.8, indicating high quantitative consistency. Our tests indicate that adding more than ten components reduces model reliability, making them less identifiable. (**A-B**) Error plots (left panel) and similarity plots (right panel) for Sucrose (**A**) and Cocaine group(**B**). Error plots illustrate the normalized reconstruction error across TCA models with varying component numbers. Each black dot corresponds to a model fit using different initial parameters, all of which produced nearly identical performance. Notably, reconstruction error showed almost no further improvement beyond ten components for both groups. The similarity plot displays the similarity score for TCA models with different component numbers. Each black dot represents the similarity of a given model to the best-fit model with the same number of components, demonstrating high repeatability for both groups.

**Figure S3: Four component model, cocaine impairs OFC temporal cognition and sequence generalization with irrelevant overlapping odors**

(**A**–**B**) Four components from TCA applied to Sucrose (**A**) and Cocaine (**B**) neuron activity are shown. Each component includes a neuron factor (left), a temporal factor spanning 72 time points across nine events (middle), and a trial factor (right). Temporal events are grouped as: 1–8 (preTrial), 9–16 (Light), 17–24 (Poke), 25–32 (Odor), 33–40 (Unpoke), 41–48 (Choice), 49–56 (Outcome), 57–64 (postTrial1), and 65–72 (postTrial2). The dataset contains eight trial types (40 trials each): trials 1–80 and 241–320 are rewarded (P1, P4), and 81–240 are unrewarded (P2, P3). In Sucrose, components 1, and 2. In Cocaine, components 1, 2, and 4 encode positive trials; 3 encode negative trials. Additionally, Sucrose components 3 and 4 relate to temporal cognition. (**C**) Mutual information (MI) between across-trial factors and trial types is lower in the Sucrose group, especially at components 1, 3 and 4, indicating weaker dependency. Two-way ANOVA confirmed a significant group difference ($F_{(1,544)} = 1074.5$, $p = 6.7 \times 10^{-131}$, $\eta_p^2 = 0.66$) and component effect ($F_{(3,544)} = 17.7$, $p = 5.2 \times 10^{-11}$, $\eta_p^2 = 0.089$). (**D**) MI between across-trial factors and time is higher in Sucrose, particularly at components 1, 2 and 4, reflecting stronger temporal encoding. ANOVA showed a significant group effect ($F_{(1,792)} = 36.5$, $p = 2.3 \times 10^{-9}$, $\eta_p^2 = 0.044$) and component effect ($F_{(3,792)} = 6.5$, $p = 2.4 \times 10^{-4}$, $\eta_p^2 = 0.024$). Error bars denote SEM.

**Figure S4: Fourteen component model, cocaine use impairs OFC function in temporal cognition and hinders generalization across odor-overlapping, task-irrelevant sequences**

(**A**–**B**) Fourteen components from TCA applied to Sucrose (**A**) and Cocaine (**B**) neural activity are shown. Each component includes a neuron factor (left), a temporal factor spanning nine events with eight time points each (middle), and a trial factor (right). Temporal segments are grouped as: preTrial (1–8), Light (9–16), Poke (17–24), Odor (25–32), Unpoke (33–40), Choice (41–48), Outcome (49–56), postTrial1 (57–64), and postTrial2 (65–72). The dataset comprises eight trial types (40 trials each): rewarded trials (1–80, 241–320; P1, P4) and unrewarded trials (81–240; P2, P3). Low-dimensional components were extracted from a 14-component model for each group. In Sucrose, components 2, 7, 8, 12 and 13 encode positive trials; components 1, 4, 10, 11 and 14 encode negative trials. In Cocaine, positive trial components are 2, 3, 5,7, 8, 11, 12, and 13; negative trials are encoded by components 1, 4, 6, 9, 10, and 14. Additionally, Sucrose components 3, 5 and 6 encode temporal structure, and component 9 is broadly active across trials. (**C**) Mutual information (MI) between across-trial factors and trial types is lower in the Sucrose group, particularly at components 3, 5 and 6, indicating weaker encoding of trial identity. A two-way ANOVA confirmed a significant group difference ($F_{(1,308)} = 571.6$, $p = 4.1 \times 10^{-25}$, $\eta_p^2 = 0.29$) and component effect ($F_{(13,308)}$ 33.0, $p = 1.0 \times 10^{-50}$, $\eta_p^2 = 0.58$). (**D**) MI between across-trial factors and time is higher in Sucrose, especially at components 5 and 9, reflecting stronger temporal encoding. A two-way ANOVA revealed a significant group effect ($F_{(1,2772)} = 3.8$, $p = 0.046$, $\eta_p^2 = 0.0014$) and component effect ($F_{(13,2772)} = 5.5$, $p = 5.6 \times 10^{-10}$, $\eta_p^2 = 0.025$). Error bars represent SEM.

## ACKNOWLEDGMENTS

This research was funded by the Intramural Research Program at the National Institute on Drug Abuse (ZIA-DA000587). The views expressed in this article are solely those of the authors and do not necessarily represent the opinions of the NIH or DHHS.

## AUTHOR CONTRIBUTIONS

J.Z. and G.S. designed the experiments, while J.Z. and W.Z. collected and analyzed the data with guidance and technical support from Z.Z and L.E.M.. W.Z., J.Z., and G.S. wrote the manuscript, incorporating feedback from all authors.

## DECLARATION OF INTERESTS

The authors declare no competing interests.

## STAR ★ METHODS

### KEY RESOURCES TABLE

In a separate file.

### CONTACT FOR REAGENT AND RESOURCE SHARING

This study did not generate any unique reagents. However, for further information or requests for resources and reagents, please contact the Lead Contact, Geoffrey Schoenbaum (geoffrey.schoenbaum@nih.gov).

### EXPERIMENTAL MODEL AND SUBJECT DETAILS

The study was conducted on a group of six male Long-Evans rats (Charles River strain), aged around three months and weighing between 175–200 g. The rats were housed individually in an AAALAC-accredited animal care facility at the National Institute on Drug Abuse Intramural Research Program (NIDA-IRP), with *ad libitum* access to food on a 12-hour light-dark cycle. Water was removed a day before testing, and the rats were provided with free access to water for 10 minutes each afternoon in their home cages. If there was no testing scheduled for the following day, they were given free access to water. All behavioral testing was conducted at the NIDA-IRP, and the animal care and experimental procedures were conducted in accordance with the guidelines set by the US National Institutes of Health (NIH) and approved by the Animal Care and Use Committee (ACUC) at the NIDA-IRP.

### METHOD DETAILS

#### Behavioral Tasks

##### Figure 8 Task

The study employed aluminum boxes (18 in. on a side) equipped with a port for odor delivery and a well for delivery of sucrose solution for conducting behavioral training. A custom-written C++ program and a system of relays and solenoid valves were used to control the task events. The entries into the odor port and the fluid well were detected by infrared beam sensors. The availability of each trial was signaled by the illumination of two house lights above the odor port. The trial was initiated if the rat entered the odor port within 5 s after light onset, leading to odor delivery after a 500-ms delay. The rats were required to remain in the port for an additional 500 ms; otherwise, the trial was aborted, and the lights extinguished. After 500 ms, the rats were free to leave the port, terminating odor delivery. Post port exit, the rats had 2 s to respond at the fluid well. Responding on rewarded trials led to the delivery of a sucrose solution (10% w/v; 50 μL) after a random delay ranging from

400 to 1,500 ms. On nonrewarded trials, nonresponding during the 2-s period, or responding after the 2 s, the house lights were extinguished, indicating the end of the trial and the beginning of the ITI. Correct trials were followed by a 4-s ITI, and trials on which the rat made an error were followed by an 8-s ITI.

The study for the Figure 8 task included six odors, organized into two sequences (S1 and S2) that occurred repeatedly in turn (S1→S2→S1→S2→…→S1→S2; 40 repeats of each sequence). The odor identity is indicated by a number, and reward and nonrewarded are indicated by the positive (+) and negative (−) symbols, respectively:

S1: 5+, 0−, 1−, 2+

S2: 3+, 0−, 1−, 4+

The rats were trained on the full set of sequences from Day 1 until they achieved >75% accuracy on every trial type in a session. Following this, electrode arrays were implanted bilaterally in the orbitofrontal cortex.

**24-Position Odor Sequence Task**

Rats were trained in the same aluminum box as in the Figure 8 task. The single-trial structure was the same expect for the organization of odor cues was. Specifically, each trial presented one of 16 odors, organized into two sequence pairs: S1a/S1b and S2a/S2b.

S1a: 0+, 1−, 4−, 5+, 6−, 7+;

S1a: 2+, 3−, 4−, 5+,6−, 7+;

S2a: 8+, 9−, 12−, 13+, 14−, 15+;

S2b: 10+, 11−, 12−, 13−, 14+, 15+.

Initially, rats were trained on a single odor pair until reaching >90% accuracy, then additional pairs were introduced until performance exceeded 75% across all trials. After mastering S1a/S1b, rats were trained on odor pair 13/14 from S2, followed by additional S2 pairs after multiple reversals. Once all sequences were incorporated, final sessions consisted of 480 trials, with S1 sequences followed by S2 sequences at roughly equal probabilities (0.55/0.45) and vice versa (0.67/0.37). Rats were trained on the full sequence set until they achieved >75% accuracy on all trial types within a session, after which electrode arrays were bilaterally implanted in the OFC.

**Surgical Procedures**

Rats were surgically implanted with a total of 32 electrodes, organized into two bundles of 16 electrodes each. These bundles were constructed using nickel-chromium wires with a bare diameter of 25 µm (AM Systems, WA). The implantation targeted the bilateral orbitofrontal cortices (AP: 3 mm, ML: 3.2 mm). To ensure proper placement, each wire bundle was encased in a 27-gauge stainless-steel tubing and trimmed using fine spring scissors. The trimmed wires extended approximately 1.5-2 mm beyond the tubing's end. Initially, the wire tips were positioned 4.2 mm ventral from the brain surface. Following the surgical procedure, the rats received oral doses of Cephalexin (15 mg/kg) twice daily for a duration of two weeks to prevent any potential infections.

**Catheter Surgery**

Rats used for cocaine self-administration received chronic indwelling jugular catheter implants (Instech Laboratories). Rats were anaesthetized using ketamine (100 mg/kg, i.p., Sigma) and xylazine (10 mg/kg, i.p., Sigma). Blunt dissection was performed to isolate right external jugular veins and catheters were surgically implanted 3 cm into the veins. Catheters were passed subcutaneously to the back, where they were attached to an external harness. Carprofen (5 mg/kg, s.c., Pfizer) was administered after surgery as an analgesic. Rats recovered for 7 days before self-administration began. During recovery and self-administration, catheters were flushed daily with a cocktail of enrofloxacin (4.0 mg/mL, Bayer) and heparinized saline (50 IU/mL in 0.9% sterile saline, Sigma) to maintain catheter patency.

**Self-Administration**

Following recovery from catheterization surgery, rats were trained to self-administer cocaine-HCl (0.75 mg/kg/infusion; $n$ = 3) or sucrose (10% wt/vol; $n$ = 3) for 14 consecutive days. Rats were trained in modular behavioral test chambers (Coulbourn Instruments) housed in sound-attenuating boxes. Each chamber was equipped with two levers positioned 8 cm above the floor on opposite sides of the same wall. For intravenous cocaine self-administration, catheter ports were attached to silastic tubing connected to infusion pumps (Med Associates Inc.) located outside sound-attenuating boxes. For sucrose self-administration, sucrose solution was delivered via photobeam-monitored recessed dippers. Daily sessions were 3 hours and began with the illumination of a house light and the insertion of an active lever. Under a fixed ratio 1 (FR1) schedule of reinforcement, active lever presses resulted in 4 s infusions or dipper insertions (0.05 mL), for cocaine-HCl or sucrose, respectively, and were paired with the illumination of a cue light above the active lever. Infusions and dipper insertions were followed by a 40 s timeout period when the active lever retracted and the house light was extinguished. Following the timeout period, the lever was reinserted and the house light was turned back on. Inactive lever presses had no programmed consequence. Reinforcers were limited to 20 *per* hour to prevent overdose in cocaine self-administering rats. When 20 reinforcers were earned in less than an hour, a timeout period as described above was imposed until the beginning of the next hour.

**Single-Unit Recording**

Electrophysiological signals were recorded using Plexon OmniPlex systems (Plexon, Dallas, TX). These signals were digitized, amplified, and subjected to bandpass filtering (250–8,000 Hz) to isolate spike activity. Manual thresholding was performed on each active channel to capture unsorted spikes. Timestamps for behavioral events were synchronized with the Plexon system and recorded together with the neural activity. To remove noise and identify single units, spike sorting was carried out offline using Offline Sorter (v.4.0; Plexon), utilizing a template-matching algorithm. The sorted files were then processed in NeuroExplorer (Nex Technologies, Colorado Springs, CO) to extract timestamps for both unit activity and behavioral events. Subsequently, these timestamps were exported as MATLAB (2021b; MathWorks) formatted files for further analysis. It is important to note that the electrodes were not advanced within a specific problem. However, we cannot make any claims regarding the consistency of single units recorded on different days within the same problem, as they may represent distinct neurons. To sample different neural populations during odor problems, the electrodes were advanced by approximately 120 μm.

### QUANTIFICATION AND STATISTICAL ANALYSES

Quantification and statistical analyses were conducted using MATLAB (R2024b; MathWorks) and Python Software Foundation, 2024. The sample sizes of rats and neurons were not predetermined through specific statistical methods; however, they are consistent with those reported in previous studies conducted by our lab and other research groups.

#### Task Events and Peri-Event Spike Train Analysis

The trials were divided into nine distinct epochs, each corresponding to different task events: "ITI-a", "Light", "Poke", "Odor", "Unpoke", "Choice", "Outcome", "postOutcome", and "ITI-b". "ITI-a" represented the time point 0.7 seconds before the house-light turned on. On reward trials, the well-entry moment was labeled "Choice." The "Outcome" epoch denoted the time of reward delivery. On non-reward trials, the end of the 2-second response window was marked as "Choice," and a time point 0.7 seconds after "Choice" was labeled as "Outcome." Both on reward and non-reward trials, 0.7 seconds after the outcome was recorded as "postOutcome," followed by another 0.7 seconds designated as "ITI-b." Behavioral performance was evaluated by calculating the percentage of trials in which the rats responded correctly and determining the latency at which they initiated a trial after the onset of the light. The spike train for each isolated single unit was aligned to the onset of each task event to create a peri-event time histogram (PETH). The PETH was constructed with a pre-event time of 200 ms and a post-event time of 600 ms, counting the number of spikes within each 100 ms bin. To smooth the PETH on each trial, a Gaussian kernel with a σ (standard deviation) of 50 ms was applied. For further analysis, a random selection of 30 correct trials was made from each trial type, resulting in a total of 240 trials (30 trials × 8 trial types). The post-event firing rates (100–600 ms) were averaged to obtain a single measure of neural activity for each neuron on each trial during each task epoch.

#### Classification Analyses

The neural data collected during each task epoch were organized into a two-dimensional matrix, where the rows represented individual trials and the columns represented the firing rates of each neuron across all trials. In other words, each trial was represented as a vector, with each dimension corresponding to the firing rate of a specific neuron. Neurons recorded across different sessions were concatenated, aligning them with the corresponding trials to create pseudoensembles. To remove temporal correlations between neurons and generate different pseudoensembles, we shuffled the trial orders within each trial type. This shuffling process was repeated 10,000 times, resulting in 10,000 pseudoensembles. By using the linear Support Vector Machine (SVM) for classification analyses, we assessed the classification accuracy through a leave-one-out cross-validation procedure. Specifically, one trial from each trial type was excluded for future testing, while the remaining trials were used to train the classifier. For each pseudoensemble, the leave-one-out cross-validation was repeated 200 times to estimate the mean decoding accuracy. The decoding analyses were conducted on the 10,000 pseudoensembles to calculate an overall mean decoding accuracy. To determine the statistical significance of the overall mean decoding accuracy, we estimated a 95% confidence interval by running the same decoding process with label-shuffled pseudoensembles.

**TCA Analysis**

To perform TCA, neuronal firing rates for each group were structured into a three-dimensional array ($N \times T \times K$), where $N$ represents the number of neurons, $T$ the time samples *per* trial, and $K$ the number of experimental trials. This array, referred to as a third-order tensor, captures neuronal activity across trials. Data were exported as a MATLAB .mat file and imported into Spyder for analysis using the TensorTools package [16]. Temporal factors were grouped as follows: factors 1–8 (preTrial), 9–16 (Light), 17–24 (Poke), 25–32 (Odor), 33–40 (Unpoke), 41–48 (Choice), 49–56 (Outcome), 57–64 (postTrial1), and 65–72 (postTrial2). There are 8 trial types, each with 40 trials. Trial factors 1–80 and 241–320 represent positive trials with reward, while 81–240 correspond to negative trials without reward. Trials 1–80 map to P1, 81–160 to P2, 161–240 to P3, and 241–320 to P4. Error and similarity plots were generated to assess the stability of TCA's optimization landscape. Specifically, we ran the TCA optimization algorithm 100 times for each of 10 components, initializing each run with random conditions, and plotted the normalized reconstruction error across all iterations. This approach allowed us to evaluate whether certain runs converged to local minima with high reconstruction errors. Additionally, similarity scores were computed for each model relative to the best-fit model with the same number of components, with the lines representing the mean similarity as a function of component count. Across all tested component numbers, the 100 repeated runs exhibited substantial overlap and consistently produced similarity scores above 0.8, demonstrating high quantitative consistency. MI was calculated for across-trial factors and trial type, as well as across-trial factors and time, after aligning components between the Sucrose and Cocaine groups. The alignment followed the same ordering of across-temporal factors between the two groups. MI was calculated for across-trial factors and trial type, as well as across-trial factors and time, after aligning components between the Sucrose and Cocaine groups. The alignment followed the same ordering of across-temporal factors between the two groups.

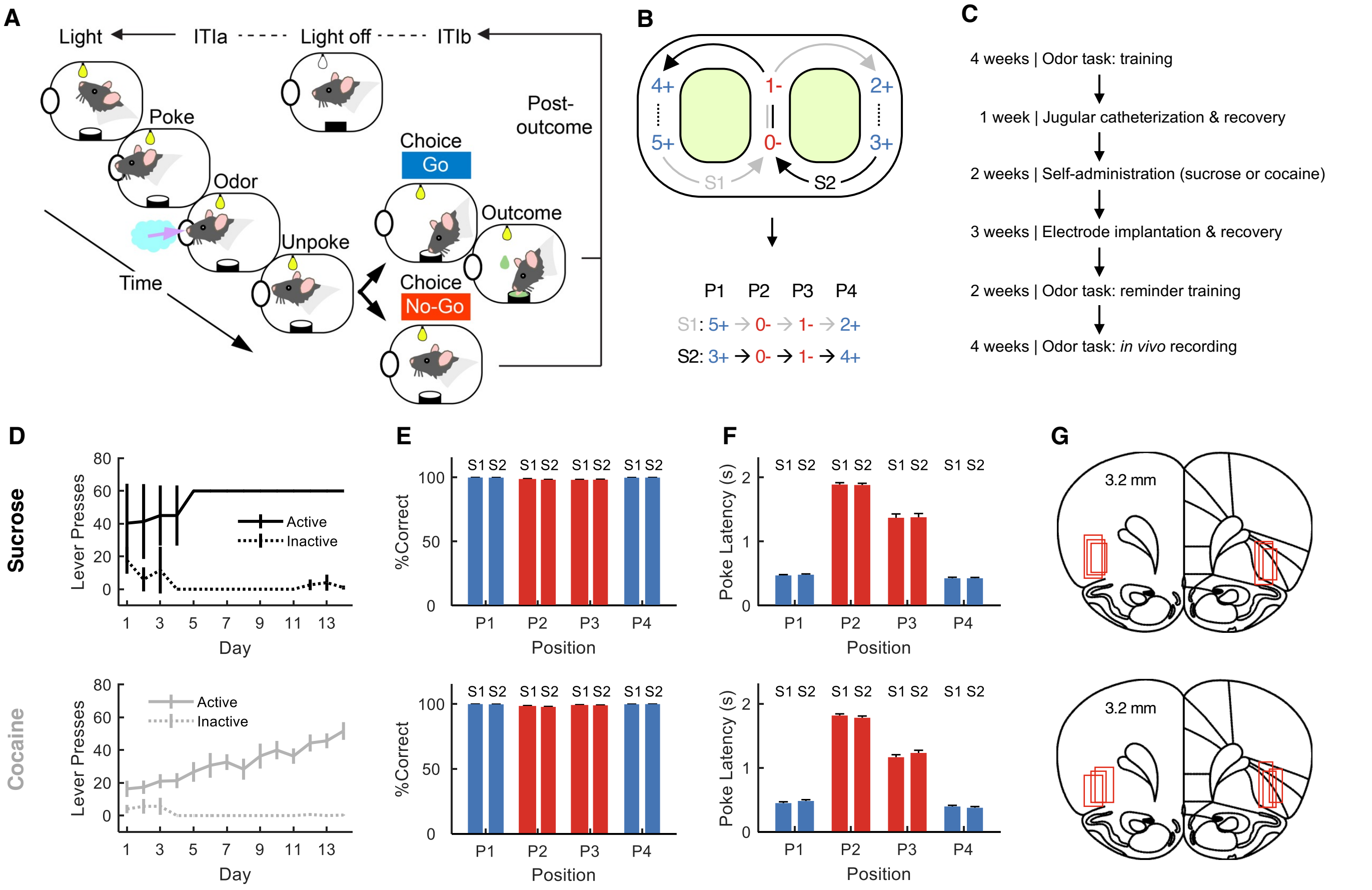

Figure 1. figure 8 odor sequence task and cocaine self-administration

[illegible]
(which was not certified by peer review) is the author/funder. This article is a US Government work. It is not subject to copyright under 17 U
105 and is also made available for use under a CC0 license.

### Single-cell selectivity

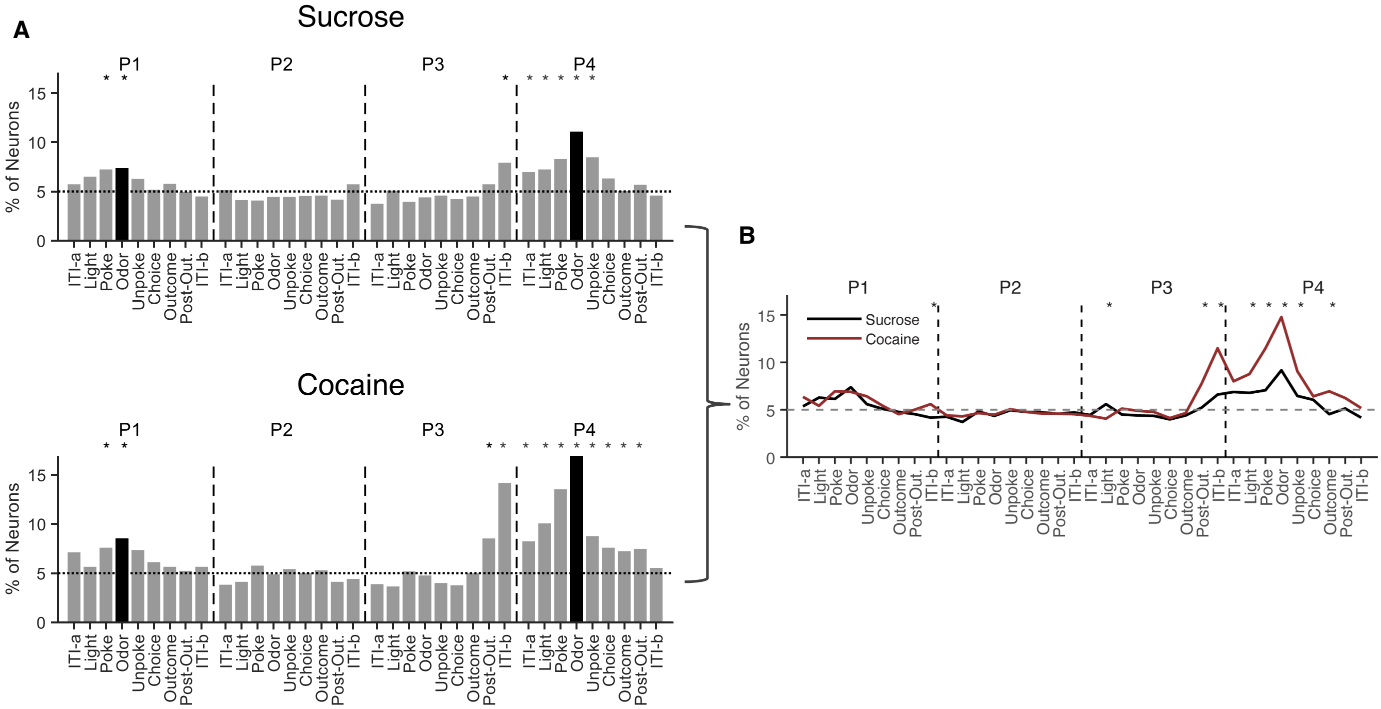

Figure 2. Cocaine use enhances the OFC's ability to discriminate between sequences S1 and S2 at single-cell level

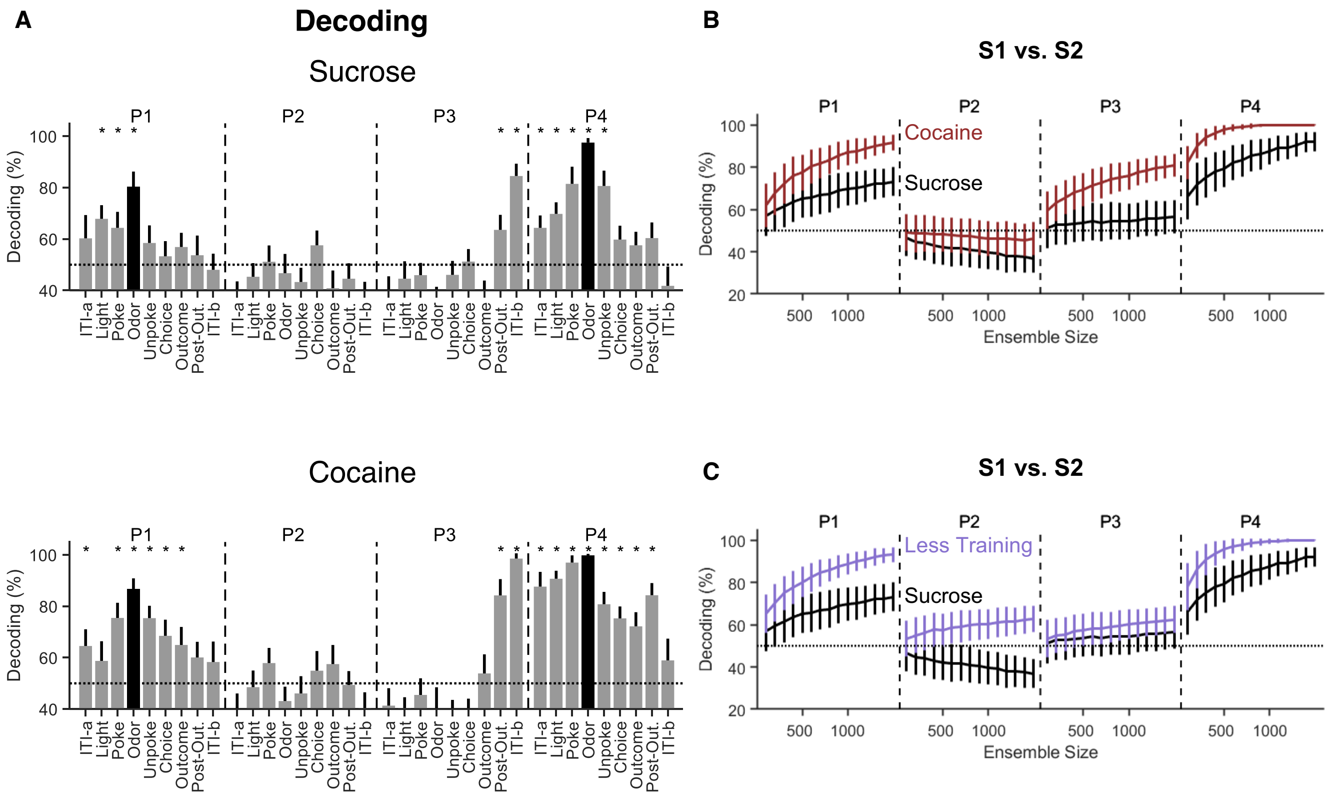

Figure 3. Cocaine use reduces the ability of OFC to generalize task-irrelevant odor-overlapping sequences

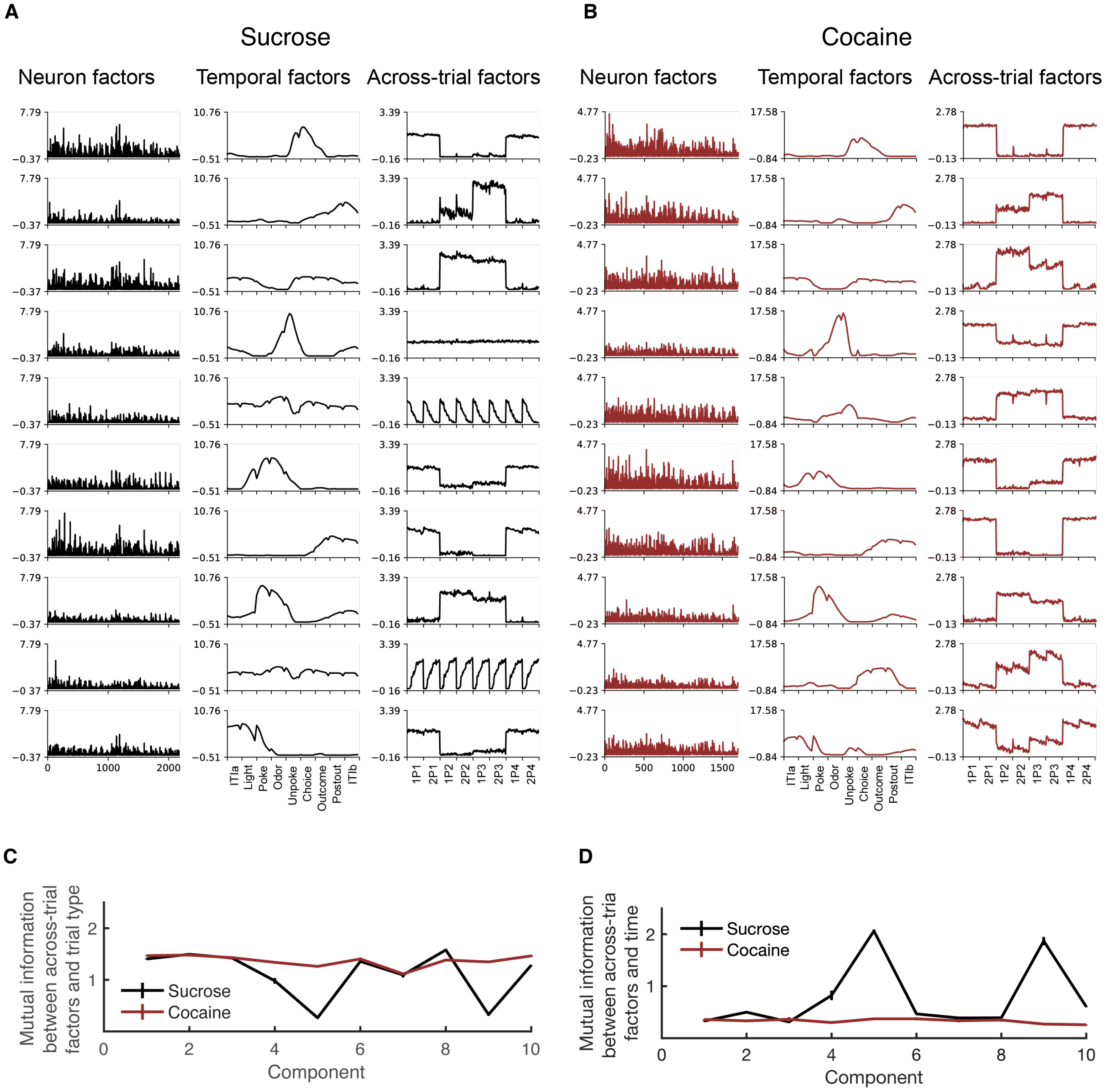

Figure 4. Cocaine use impairs the OFC's function in temporal cognition and decreases the generalization of task-irrelevant odor-overlapping sequences

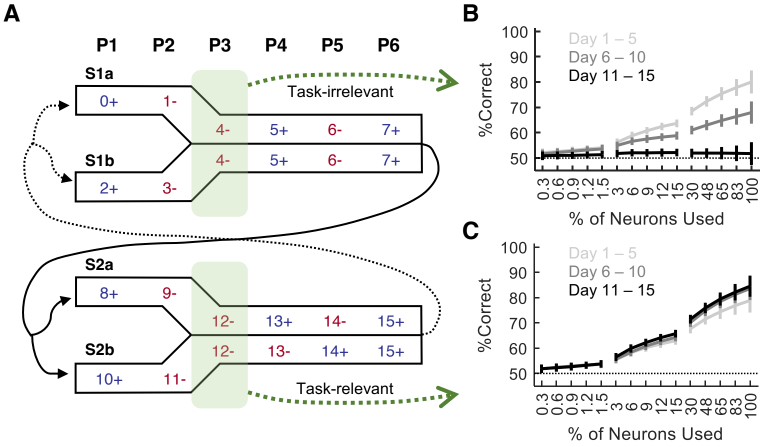

Figure S1. The ability of the OFC to generalize task-irrelevant odor-overlapping sequences decreases as learning progresses

Figure S2. Error and similarity plots for TCA

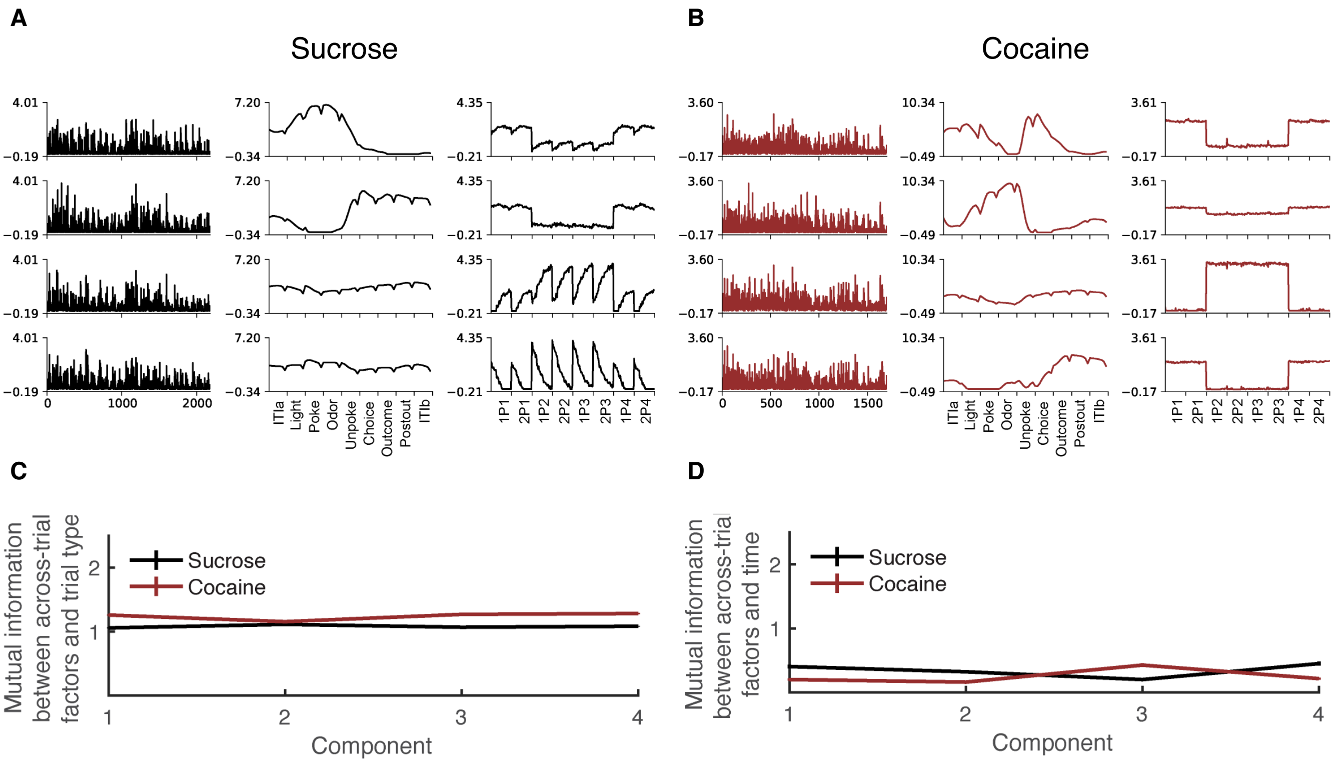

Figure S3. Four component model, cocaine impairs OFC temporal cognition and sequence generalization with irrelevant overlapping odors

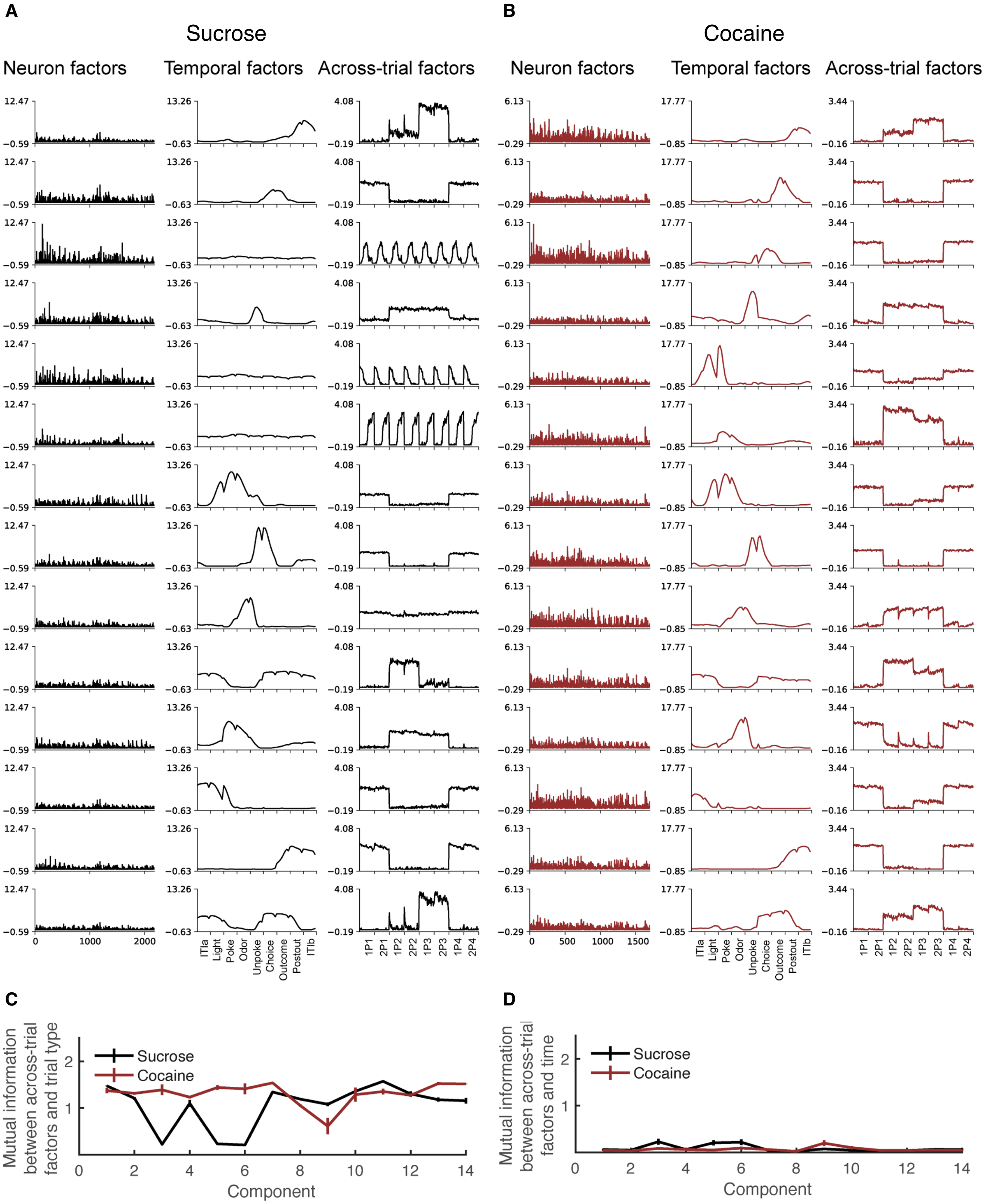

Figure S4. Fourteen component model, cocaine use impairs OFC function in temporal cognition and hinders generalization across odor-overlapping, task-irrelevant sequences
